# The dis-economy of precision medicine: how stratification multiplies social tension in healthcare systems

**DOI:** 10.1007/s11019-026-10381-z

**Published:** 2026-07-11

**Authors:** Raef Kozman, Fabrice Jotterand

**Affiliations:** 1 Institute of Biomedical Ethics, Basel University, Bernoullistrasse 28 - 2nd Floor, 4056 Basel, Switzerland; 2 Oregon Health & Science University Center for Ethics in Health Care, Portland, OR US

**Keywords:** Precision medicine, Healthcare systems, Economics, Cost-effectiveness

## Abstract

Precision medicine (PM) promises better outcomes by aligning prevention and treatment with molecular and phenotypic profiles. But precision is not merely a clinical refinement; it is an epistemic shift in how health systems classify risk, allocate eligibility and define value. In this study, we argue that broad adoption of PM creates structural tension within solidarity-based health systems and exposes limits in conventional cost-effectiveness evaluation. Ethically, PM converts pooled probabilistic uncertainty into individualized biological prediction, making access increasingly conditional on measurement, interpretation and pathway completion. Economically, PM fragments evaluable populations, destabilizes counterfactual comparison and shifts value from discrete products to information-dependent care pathways. As a result, standard metrics such as the incremental cost-effectiveness ratio (ICER) often become sensitive not only to biological effect, but also to governance: who is tested, how results are interpreted, which downstream services are funded and who can complete the pathway. We suggest that the instability observed in PM appraisal is not simply a technical problem of modeling, but a sign that the object of evaluation has changed. Recognizing this structural shift is essential if PM is to be integrated into health systems committed to both equity and evaluability.

## Introduction: precision as an epistemic shift

Precision medicine has become a defining ambition of contemporary healthcare (Muharremi et al. 2023). Genomic sequencing, molecular diagnostics and AI-supported analytics promise earlier intervention, better alignment between therapy and mechanism, and reduced exposure to ineffective care (Jameson and Longo 2015). Yet precision is not only a technological advance. It reorganizes how health systems understand risk (Hersey et al. 2026). Although the paper focuses on genomic and molecular PM as the paradigmatic case, the argument is not limited to genomic stratification. Similar pressures arise when risk is inferred from imaging, electronic health records, environmental exposure, food consumption, occupation, education, postal code, race, ethnicity or other social and behavioral data. The common feature is not the biological source of the data alone, but the conversion of heterogeneous persons into increasingly granular risk categories. AI-supported analytics intensify this process by allowing multiple data layers to be combined and used for prediction, triage, eligibility or prioritization (Marques et al. 2024, Zhang et al. 2025). Genomic PM is therefore treated here as a particularly visible case of a broader movement toward datafied stratification in healthcare.

Solidarity-based systems were built on pooled uncertainty (Cronk and Aktipis 2021). Insurance remains politically and ethically defensible because future illness is only partly knowable, risks are aggregated and redistribution occurs across heterogeneous populations (Scordis and Pompella 2025, RUGER JP 2007). Health economic evaluation developed within the same architecture, comparing interventions across relatively stable populations and summarizing incremental value in population-based metrics (Mosadeghrad et al. 2022).

PM alters these conditions. By rendering biological heterogeneity measurable and clinically actionable, it shifts medicine from pooled probabilistic uncertainty toward individualized prediction (Marques et al. 2024). This transformation has ethical consequences for access, eligibility and solidarity, and economic consequences for how value is measured and justified (Brothers and Rothstein 2015, Gavan et al. 2018).PM can help clinicians identify which patients are more likely to benefit from a specific intervention, avoid ineffective treatment, or receive earlier diagnosis. Yet it also changes the route through which patients become eligible for care. A patient may need to be tested, have results interpreted, be referred to a specialist pathway, and have access to the relevant downstream intervention before the promised benefit of precision medicine can be realized. Patients who cannot enter or complete this pathway may remain in standard care, even when their biology would have made them candidates for more targeted intervention.

This paper argues that this shift matters for both ethics and evaluation. Ethically, PM can make access increasingly dependent on whether a person is measured, classified and made visible to the system. Economically, it can make the value of an intervention depend not only on the intervention itself, but also on who receives testing, how results are interpreted, which follow-up services are available and whether patients can complete the pathway. The central claim is therefore not that precision medicine is clinically undesirable. Rather, it is that precision medicine must be understood as a governed care pathway, not merely as a more accurate biological technology.

The argument proceeds in four steps. First, we examine how PM shifts health systems from pooled uncertainty toward individualized prediction. Second, we show how this shift can affect insurance, eligibility and biological visibility. Third, we explain why conventional economic evaluation becomes less stable when interventions are organized around increasingly narrow and pathway-dependent subgroups. Finally, we argue that the future challenge for universal health care is not whether precision medicine will advance, but whether it can be integrated without weakening solidarity, equity and public evaluability.

## From pooled uncertainty to biological prediction

Insurance is a technology of uncertainty management (Parsons 2015). In solidarity-based arrangements, individuals contribute to collective pools without precise knowledge of their future health trajectory (Chris James 2010, Peter and Smith 2004, ChenG T 2020). That shared ignorance helps sustain redistribution from lower-risk to higher-risk individuals (Baeza and Packard 2006, Roos and Schut 2012).

PM reduces this shared ignorance. Genetic testing can identify predisposition before symptoms emerge; pharmacogenomics can predict drug response; tumor profiling can redefine treatment eligibility (Ventola 2013, Roden et al. 2011, Alliance 2009, Tjota et al. 2024). What was once a population-level probability becomes a personal biological attribute. This does not mean that solidarity-based systems must collapse into actuarial sorting. It does mean, however, that PM creates a persistent tension between redistributive justice—the commitment to pool resources and allocate care according to need across the population and the growing technical capacity to differentiate individuals by predicted risk and likely benefit. The literature on precision medicine and solidarity has warned that such differentiation can weaken shared risk-pooling by making ever finer distinctions between who is expected to benefit, at what cost, and with what priority (Fleck 2022, Green et al. 2023). At the same time, because access to testing, data infrastructures, and specialist interpretation is uneven, precision approaches may also reproduce structural inequities rather than merely reveal biological difference. That tension cannot be dismissed by assumption; it must be governed.

This analysis extends existing debates on the economic evaluation of precision medicine by showing that the problem is not only methodological but structural. The same stratifying logic that improves biological targeting also fragments evaluable populations, complicates counterfactual comparison, and redistributes access to benefit through data availability, interpretability, and pathway capacity.

## Insurance as an illustration: private markets and public systems

The challenge is both technical and ethical. It is technical because PM creates smaller subgroups, more complex pathways, thinner evidence, unstable comparators and greater uncertainty in economic evaluation (Mandelblatt et al. 1997, Kasztura et al. 2019, Chen et al. 2023). But it is also ethical because these technical features affect who is recognized as eligible, whose needs are made visible, whose risk is pooled and whose claim on shared resources is judged worthwhile. A purely technical framing would suggest that the problem can be solved by better models alone. Better models are necessary, but insufficient. The deeper normative issue is whether a solidaristic health system can use more precise predictive information without allowing that information to erode equal concern, pooled protection and access according to need.

Private insurance markets are useful for the present argument because they make the incentive structure of risk differentiation visible. They do not represent the endpoint of solidarity-based public systems. Rather, they illustrate what happens when increasingly precise information about future health risk becomes actionable (Layton et al. 2017, Greß 2007). Where genetic or predictive information can be used in underwriting, incentives arise to divide people into finer risk categories, charge different premiums, exclude some individuals or narrow the scope of coverage. Where such use is prohibited, pooling is sustained by regulatory constraint rather than by ignorance (Prince 2017, Prince 2019, Hall and Rich 2000). Public and universal systems usually do not engage in individual underwriting in the same way, but analogous segmentation can reappear through clinical and reimbursement design: who is offered testing, who meets biomarker or referral thresholds, who qualifies for targeted therapy, and who remains in standard care. The policy problem is therefore not limited to private insurance law. It concerns how differentiation re-enters systems that are formally committed to pooled entitlement.

Exclusion in PM need not occur through explicit denial of coverage. It can be reproduced through institutional rules that differentially distribute diagnostic access, therapeutic eligibility and the capacity to benefit from precision care.

## Biological visibility and the digital divide

PM is not simply a therapeutic advance; its clinical implementation depends on a wider socio-technical infrastructure, including sequencing platforms, laboratory quality, bioinformatics capacity, genetic counseling, interoperable health records and, increasingly, algorithmic or computerized clinical decision support (Edsjö et al. 2023, Abul-Husn and Kenny 2019).

Access to PM is therefore mediated not only by disease biology, but also by health-system capacity: specialized infrastructure, trained workforce, interoperable data systems, and governance arrangements that allow genomic information to be used safely and effectively in routine care. It is further shaped by digital inclusion and socioeconomic position. In this setting, inequality appears not merely as a treatment gap, but as a gradient of biological visibility: a difference in the extent to which patients’ molecular characteristics are identified, interpreted and incorporated into clinical decision-making. Patients who undergo molecular characterization become legible to contemporary care pathways in ways that others do not. They may qualify for targeted therapies, intensified surveillance, cascade testing or preventive interventions unavailable to those who are not profiled. Those outside the precision pathway remain governed by broader statistical averages, even when their biology differs materially from the population’s mean.

This is why PM can act as an inequality multiplier. When access to testing, digital tools and specialist referral is uneven, the precision layer tends to accumulate around already advantaged groups, while underserved populations remain in conventional pathways, enter later or never enter at all. The ethical problem is therefore not only who receives the test. It is that unequal access to measurement reconstitutes eligibility itself. Once biological visibility becomes selective, the boundary between an access problem and an evaluation problem begins to dissolve, because what is being evaluated now depends on who is made visible to the system in the first place. This matters not only for justice, but also for evaluation. If access to molecular visibility is uneven, then the population entering precision care is not simply discovered; it is socially and institutionally produced. Under those conditions, the basic inputs of conventional cost-effectiveness analysis—who is eligible, compared with whom, and on what evidentiary basis—can no longer be treated as stable.

This creates a precision paradox. Access to PM can benefit patients by producing a more accurate diagnosis, avoiding ineffective treatment, reducing adverse events and enabling targeted therapy. Yet the same predictive knowledge can expose patients to new forms of disadvantage if it is used outside a protected clinical context. A patient may become more clinically visible and at the same time more administratively vulnerable: visible as high risk, costly, unlikely to benefit, or outside a reimbursed pathway. In poorly governed systems, not knowing may preserve access to pooled-risk protection, while knowing may produce exclusion or conditional entitlement. The ethical problem is therefore not access to PM as such, but the institutional use of predictive information. PM is emancipatory when it expands the capacity to care; it is exclusionary when prediction becomes a basis for denying, delaying or narrowing access.

## Economic evaluation under stratification: why cost-effectiveness becomes governance-sensitive

Before turning to the specific challenge posed by PM, it is useful to clarify the economic terms used in this section. Health economic evaluation refers to the set of methods used to compare the expected costs and consequences of alternative health interventions. Cost-effectiveness analysis asks whether the additional health benefit of an intervention is worth its additional cost, often expressed as an incremental cost-effectiveness ratio, or ICER: the extra cost required to gain one additional unit of health benefit, such as a quality-adjusted life year, or QALY. Budget impact analysis asks a different but related question: whether the total cost of adopting an intervention is affordable for a given health system. Opportunity cost refers to the health benefits that may be lost elsewhere when scarce resources are allocated to one intervention rather than another (Wouterse et al. 2023, Phelps 2024, Palmer et al. 1999). These concepts are not merely technical. In solidarity-based systems, they help determine which claims on shared resources are publicly justified.

Conventional cost-effectiveness analysis was developed for relatively bounded interventions: a drug, a procedure or a screening programme with stable eligibility rules and a reasonably well-defined comparator (Mandelblatt et al. 1997, Economic Evaluation 2026). PM places pressure on these assumptions. First, it fragments evaluable populations into increasingly narrow subgroups defined by molecular features. As biomarker prevalence falls, empirical evidence becomes thinner and uncertainty rises. Second, PM is rarely a single intervention. It is more accurately understood as a linked pathway consisting of testing, interpretation, counseling, eligibility assignment, treatment choice, monitoring and, in some cases, family-level or cascade effects. Third, the relevant comparison is often no longer “drug versus no drug” or even “test versus no test,” but one evolving information regime versus another.

Under these conditions, the ICER becomes sensitive not only to biology, but also to governance. It begins to reflect institutional design choices — sequencing strategy, reimbursement rules, referral architecture, implementation quality and downstream capacity — as much as the intrinsic performance of the technology itself. The applied literature is consistent with this fragility: reported value-for-money estimates for precision medicine often vary with biomarker prevalence, comparator choice, time horizon, pathway specification, and study perspective, rather than yielding a readily stable or transferable estimate of value (Zhang et al. 2025, Kasztura et al. 2019, Chen et al. 2023). The central problem is therefore not merely statistical imprecision. It is that stratification changes the object of evaluation. Once value depends on who is tested, how results are interpreted and what follow-up the system can sustain, the ICER ceases to function as a neutral summary statistic of a fixed intervention. It becomes a contingent measure of a governed pathway.

## The N-of-1 regulatory crisis: when biological precision exceeds universalism

PM challenges appraisal not chiefly because it is costly, but because it increases resolution. As biomarkers proliferate, eligible populations fragment into ever smaller molecularly defined strata. In the limit, PM approaches an N-of-1 logic: a treatment rule valid only for a narrowly specified biological profile, sometimes approaching patient uniqueness (Wang and Wang 2023, Samuel et al. 2023, Kim-McManus et al. 2024). This creates a structural mismatch with Universal Health Coverage (UHC).

UHC is built on pooled risk and broad population coverage, while conventional health-economic evaluation relies on comparability across persons through generic outcome measures (Abiiro and Allegri 2015, Tulchinsky 2018). Its ethical and economic coherence depends on administratively tractable populations, relatively stable benefit packages and collective justification at the population level (Kutzin 2013, Alwan et al. 2023, Lal 2026). PM moves in the opposite direction. It replaces broad comparability with fine-grained stratification, pooled uncertainty with biologically informed selection and standardized pathways with sequential, information-dependent care. UHC universalizes access by abstraction, grouping persons into broad categories of entitlement, whereas PM increasingly conditions access by differentiation, sorting patients according to ever more specific biological distinctions.

This contradiction becomes most visible in Health Technology Assessment (HTA). Conventional appraisal assumes target populations that can be delimited with reasonable stability, evidence capable of estimating average effects with tolerable uncertainty and outcomes that can be summarized in decision-grade metrics (Briggs and Gray 1999, Vreman et al. 2020). PM progressively weakens each premise. The problem is deeper than wide confidence intervals. Under PM, the intervention is often not a product but a pathway architecture, and pathway design, subgroup definition and time horizon help constitute the intervention itself. Precision can therefore improve outcomes for selected individuals while reducing population-level net benefit if coverage breadth, pathway capacity and budget impact are not explicitly governed (Gavan et al. 2018, Wright et al. 2022, Wu et al. 2021, Leelahavarong 2014). That possibility is not accidental. It follows from a structural tension: precision medicine tends to allocate access and therapeutic eligibility through biological stratification and data-dependent clinical visibility, whereas UHC is organized around membership, pooled risk, and solidarity-based access according to need.

The epistemic challenge compounds the distributive one. In PM, the comparator is rarely a clean “no-genomics” world. Once molecular information is known, reconstructing the untested state becomes difficult. The counterfactual is therefore not simply an alternative treatment, but an alternative regime of information, uncertainty and clinical action. This is why the N-of-1 crisis is not merely an evidence problem. It reflects a conflict between two organizing principles of medicine: universalism through pooled entitlement, and precision through selective stratification.

## Segmentation diseconomies: when precision raises per-patient evidence and screening costs

By “segmentation diseconomies,” we do not mean that PM is economically wasteful in every case. Many precision interventions can be clinically beneficial and, under specific conditions, cost-effective. The term refers instead to system-level costs and frictions that increase as stratification becomes finer. A health system may need to test many patients to identify a small eligible subgroup; generate evidence for increasingly narrow populations; maintain specialized laboratory, informatics and counseling capacity; and finance downstream pathways that only some patients can complete. Thus, even when the unit cost of sequencing falls, the total cost of finding, interpreting and acting on rare actionable distinctions may rise. The dis-economy lies in the loss of scale, pooling and evaluative stability that occurs when healthcare is reorganized around increasingly small and information-dependent subgroups.

PM does not eliminate economies of scale; it increasingly converts them into segmentation diseconomies. As biomarker-defined populations shrink and precision pathways proliferate, the cost per correctly identified and treated eligible patient can increase despite falling sequencing costs, because lower biomarker prevalence and greater pathway complexity raise screening, validation, and implementation burdens (Faulkner et al. 2020, Beckmann and Lew 2016, Christofyllakis et al. 2022). The burden shifts from the price of a single test to the cumulative cost of search: finding rare matches, interpreting uncertain variants, funding counseling and sustaining the downstream pathway required to translate molecular distinction into clinical benefit.

These costs do not contract in proportion to the eligible population. As stratification deepens, the denominator shrinks while the evidentiary and organizational burden remains high. Reported cost-effectiveness therefore depends less on a stable intervention effect than on the service architecture required to make that effect reachable. Precision does not merely personalize treatment; it raises the per-patient cost of evidence, coordination and delivery.

### Conclusion: precision requires governance to remain equitable and evaluable

PM improves targeting by shifting from pooled probabilistic risk toward biologically informed prediction. Yet this epistemic advance also reorders access, eligibility, and value: ethically, it can intensify digital and biological stratification by making benefit increasingly conditional on being measured, clinically interpretable, and able to traverse the full precision-care pathway.

Economically, it fragments evaluable populations, destabilizes counterfactuals and weakens the assumptions that allow value to be summarized in population-based metrics.

These pressures share a common structural source: the shift from pooled comparison to individualized stratification. This is why the tension between PM and UHC is not incidental (Green et al. 2023). UHC is financed and justified through pooled membership, broad comparability and solidarity across persons. PM increasingly allocates benefit through differential measurability, referral, classification and pathway completion.

Without explicit governance of upstream access, data inclusion, pathway capacity and budget impact, the gains of precision are likely to concentrate among those already best positioned to enter and complete the pathway, while others remain statistically averaged and clinically delayed.

Governance cannot restore the older condition of shared ignorance once predictive knowledge exists. In that sense, the “genie” cannot be put back into the bottle. But this does not make governance futile. Governance determines how predictive knowledge is institutionally used. It can prohibit or restrict the use of genomic and predictive information for exclusionary insurance practices; guarantee equitable access to testing and counseling; require that precision pathways include referral, interpretation and follow-up capacity rather than only reimbursement for the test itself; incorporate budget impact, capacity constraints and distributional effects into appraisal; and ensure that PM is evaluated as a governed care pathway rather than as an isolated technology (Wright et al. 2022, Wu et al. 2021, Leelahavarong 2014, Faulkner et al. 2020).

Governance is therefore not a later administrative layer added after PM has been evaluated. It is constitutive of what PM becomes in practice. Governance determines who is tested, how data are interpreted, which results create entitlement, what downstream services are funded, and whether patients have the practical capacity to complete the pathway.

For that reason, the task is not to choose between precision and solidarity. It is to prevent precision from becoming a segmentation technology detached from solidarity.

The technological future of PM is likely secure. It is supported by scientific momentum, commercial investment, and real clinical benefit for selected patients. The more fragile question concerns the future of universal health care under conditions of increasing biological and data-driven stratification. If health systems reimburse tests without ensuring equitable access to interpretation, referral, counselling, and follow-up, PM may advance while universal health care becomes thinner in practice. The relevant question is therefore not whether PM will continue, but what kind of health system it will help produce. Its integration into universal and solidarity-based systems will depend on governance that protects equitable access, restricts exclusionary uses of predictive information, incorporates capacity and budget impact into appraisal, and evaluates PM as a complete care pathway rather than as an isolated technology. The task is not to choose between precision and solidarity, but to prevent precision from becoming a segmentation technology detached from solidarity.
